# Prostate Cancer Disparities Between Public and Private Healthcare Patients in Tasmania, a Regional State of Australia

**DOI:** 10.3390/cancers18010079

**Published:** 2025-12-26

**Authors:** Georgea R. Foley, C. Leigh Blizzard, Marketa Skala, Frank Redwig, Jessica Roydhouse, Joanne L. Dickinson, Liesel M. FitzGerald

**Affiliations:** 1 Menzies Institute for Medical Research, University of Tasmania, Hobart, TAS 7001, Australia or georgea.foley@ki.se (G.R.F.); leigh.blizzard@utas.edu.au (C.L.B.); jessica.roydhouse@utas.edu.au (J.R.); jo.dickinson@utas.edu.au (J.L.D.); 2 Department of Medical Epidemiology and Biostatistics, Karolinska Institutet, 171 77 Stockholm, Sweden; 3 WP Holman Clinic, Royal Hobart Hospital, Hobart, TAS 7000, Australia; marketa.skala@ths.tas.gov.au; 4 Urology Department, Royal Hobart Hospital, Hobart, TAS 7000, Australia; frank.redwig@ths.tas.gov.au

**Keywords:** prostate cancer, regional care, public health, private health

## Abstract

**Background**: Prostate cancer (PrCa) outcomes are inferior in regional and rural areas compared to metropolitan centres. We evaluated patterns of care in PrCa patients treated in public and private healthcare facilities in regional Tasmania. **Methods**: This retrospective study used clinicopathological data for 2180 PrCa patients diagnosed between 2015–2022. Descriptive statistics and regression analyses determined associations between treatment facility (public vs. private) and diagnostic and treatment factors. **Results**: A significantly greater proportion of public patients were from outer regional/remote areas (prevalence ratio (PR) = 1.25, 95% CI: 1.19–1.31), presented with higher-risk disease (PR = 1.56, 95% CI: 1.22–2.00) and underwent active treatment compared to private patients (PR = 1.07, 95% CI: 1.03–1.11). Men treated privately were most likely to have low-risk PrCa (*p* < 0.001) and be managed with active surveillance (AS, 52.9%). When stratified by disease risk, public patients with intermediate (*p* < 0.001) or very high-risk/metastatic disease (*p* = 0.003) were still significantly more likely to receive active treatment than private patients. Furthermore, except for very high-risk/metastatic patients, public patients took significantly longer to commence treatment, ranging between a mean difference of 40 to 59 days depending on risk category. **Conclusions**: In Tasmania, treatment pathways for PrCa patients differ significantly between public and private healthcare sectors and may contribute to poorer outcomes in regional and remote areas.

## Simple Summary

Tasmania is a fully regional state of Australia, with the highest rate of prostate cancer-specific mortality in the country. Tasmanian prostate cancer patients may be treated in public or private healthcare institutions. A range of treatments are available, each associated with advantages and disadvantages. We found significant differences between Tasmanian prostate cancer patients treated in the public and private healthcare systems in terms of diagnostic characteristics, likelihood of undergoing active treatment, treatment type, and time to commence treatment. Our findings have diverse implications for both clinicians and patients, showing that further investigation into treatment access and equity is needed.

## 1. Introduction

A broad range of treatments for prostate cancer (PrCa) are available, including surgical approaches, pharmaceutical and radiation therapies, as well as watchful waiting and active surveillance (WW/AS) management approaches. Further, many patients undergo multiple different treatments throughout their disease course, whether concurrently or sequentially, with treatment plans evolving as patient priorities shift and/or disease progresses. As active PrCa therapies each carry significant side effects, including urinary, bowel, and sexual function issues, clinicians and patients must be equipped to make informed treatment choices, striking a balance that avoids over-treatment while ensuring patients receive appropriate care [1,2,3]. There are also both advantages and disadvantages associated with WW/AS approaches, with the benefit of avoiding side effects associated with active treatment counterbalanced by the potential for patient anxiety and the progression of symptoms and disease [4,5,6]. It is essential that patients not only have access to a range of potential therapies, but that they understand the risks and benefits of each option.

The Australian healthcare system comprises two sectors, public and private. Evidence suggests that treatment approaches for PrCa patients differ depending on whether they attend a public or private healthcare facility. In 2020, te Marvelde et al. examined treatment choices for men with localised PrCa [7], utilising data from the Victorian Cancer Registry. The study compared the proportions of men in private and public health services receiving radical prostatectomies (RP), with or without radiotherapy, to those undergoing curative external beam radiotherapy (EBRT) only, in the 12 months post-diagnosis. The authors concluded that there was a significant difference between the two groups, and that RP was more frequent for men diagnosed in private health services, whereas curative EBRT was less frequent for these individuals [7].

To date, no study has investigated patterns in treatment approaches in regional areas of Australia. Here, our objective was to evaluate population-based registry data from the Prostate Cancer Outcomes Registry-Tasmania (PCOR-TAS) to determine whether patterns of care differ in the fully regional state of Tasmania, specifically, to ascertain whether Tasmanian patients undergoing PrCa treatment in public and private healthcare facilities differed demographically, in their disease state at diagnosis, and in their chosen treatment paths. The results of this study may provide insight into why Tasmania has the highest age-standardised PrCa mortality rate in Australia [8].

## 2. Materials and Methods

### 2.1. Prostate Cancer Outcomes Registry-Tasmania (PCOR-TAS)

The PCOR-TAS has been described previously [9,10]. An opt out consent process was employed for PCOR-TAS participants unless a man had died before consent could be sought, in which case a waiver of consent was obtained for clinical data collection. Living PCOR-TAS participants consented to their data being used for ethically approved research. Ethics approval for this study was obtained from the Tasmanian Health and Medical Human Research Ethics Committee, Australia (project ID: 32126) and was performed in accordance with the principles of the Declaration of Helsinki. As of 31 December 2022, 3362 Tasmanian PrCa patients had been recruited into the registry (opt out rate ~5%), with corresponding PrCa diagnosis and treatment data. As not all variables were available for all participants, numbers vary between analyses.

### 2.2. Data Extraction

**Figure 1:**
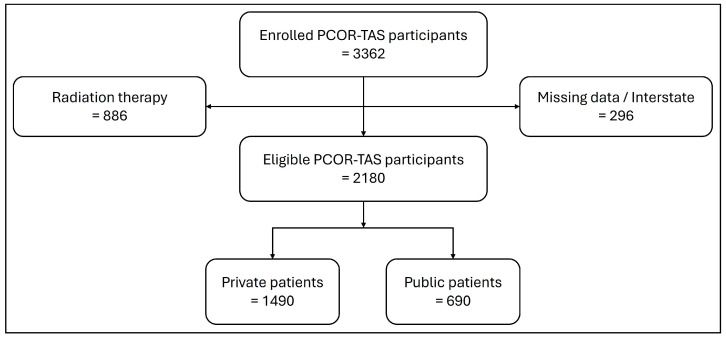
Breakdown of PCOR-TAS study participants.

De-identified, record unit level data were extracted from PCOR-TAS for the 3362 enrolled PrCa cases. Due to the current structure of the Tasmanian healthcare system and the mode of radiotherapy data capture, it was not possible to differentiate between individuals undergoing EBRT at a public vs. private healthcare facility. Therefore, individuals who underwent EBRT as a primary treatment, either alone or in combination with other treatments were excluded. Standard data cleaning and quality control checks were then undertaken, where men who were treated interstate or had missing or incomplete institution and/or treatment data were removed (Figure 1).

### 2.3. Variable Categorisation

Tasmania’s relatively centralised healthcare system is concentrated around urban hubs (classified here as inner-regional areas), with four public hospitals and nine private healthcare facilities spread across the state within these regions. Treatment facilities were classified according to their public or private status. PCOR-TAS participants were grouped according to age at diagnosis, Socio-Economic Indexes for Areas (SEIFA) category, area of residence (inner regional vs. outer regional/remote), method of diagnosis, diagnostic PSA level, diagnostic Gleason score, and risk category at diagnosis, as described previously [9]. SEIFA categories (grouped as quintiles) were calculated using the Index of Relative Socio-Economic Advantage and Disadvantage and based on postcodes (Supplementary Figure S1). Risk categories were calculated by PCOR-TAS based on National Comprehensive Cancer Network (NCCN) classifications [11] and data available to the registry (Supplementary Table S1).

Primary treatment recorded within 12 months of diagnosis was categorised as follows:

1. Active Surveillance (AS).
2. Chemical Androgen Deprivation Therapy (ADT).
3. Surgical ADT.
4. Brachytherapy.
5. Brachytherapy + ADT (Chemical).
6. Chemotherapy.
7. Chemotherapy + ADT (Chemical).
8. Focal Gland Ablation Therapy.
9. Radical Prostatectomy (RP).
10. RP + ADT (Chemical).
11. RP + Chemotherapy + ADT (Chemical).
12. Watchful Waiting (WW).
13. WW/AS Unspecified.
14. Missing.

Active treatment was defined as all of the above treatments other than AS, WW, or WW/AS unspecified. Individuals who received an active treatment after WW or AS and within twelve months post-diagnosis were considered to have received active treatment. Individuals’ time to treatment was calculated using the number of days between date of diagnosis and the date of first recorded active treatment.

### 2.4. Statistical Analyses

Demographic and clinical factor distributions are expressed as percentages and frequencies for individuals undergoing primary treatment in public or private healthcare facilities. Consistent with our primary hypothesis, chi-squared tests of independence were applied to examine differences in distributions. *p*-values less than 0.05 were considered statistically significant.

Linear (continuous variables), log binomial (binary variables) and log multinomial (categorial variables with more than two attributes) regression analyses were undertaken with demographic and clinical factors as outcome variables and treatment in a public or private institution as the explanatory factor, as described previously [9]. Results were considered statistically significant if a 95% confidence interval did not contain the null effect. All analyses were performed using Stata/SE 16.1 (StataCorp, College Station, TX, USA).

## 3. Results

A total of 3362 individuals with PrCa were enrolled in PCOR-TAS between 1 February 2015 and 31 December 2022. Of these, 2180 were eligible for analyses after removing men treated with radiotherapy (n = 886) or those missing institution or treatment data (n = 296; see Section 2.1 and Figure 1). Participant characteristics for men treated in solely public or solely private facilities (n = 2180) are presented in Table 1. Apart from categorised age at diagnosis, the publicly and privately treated groups differed significantly in the distribution of all other demographic and clinical characteristics, including median age at diagnosis, SEIFA category, disease risk category at diagnosis, and primary treatment approach.

Table 2 compares demographic and clinical variables for men treated in public and private health facilities, reported as prevalence ratios with 95% confidence intervals. A greater proportion of PrCa patients were treated in the public system compared with the private system among those from the lower end of the SEIFA distribution, those residing in outer regional and remote areas, those diagnosed via TRUS, those with the highest PSA levels at diagnosis, those with Gleason scores of 7 (4 + 3) or above, those with high-risk and very high-risk/metastatic disease, and those who underwent active treatment within the first twelve months post-diagnosis. These elevations in prevalence persisted after adjustment for age at diagnosis and after adjustment for age at diagnosis, SEIFA index, residence and method of diagnosis, except for men diagnosed with a moderately high PSA level (10.1–20 ng/mL). Interestingly, including risk category in the model attenuated the association with active treatment, however it still remained significant. The proportion of PrCa patients treated in the private system exceeded the proportion diagnosed in the public system among those in the oldest age category (≥75), highest SEIFA quintiles, those diagnosed via TP biopsy, those with lower PSA concentrations at diagnosis (4.01–10 ng/mL), those with a Gleason score less than 7, and those with low-risk disease at diagnosis. These elevations in prevalence also persisted after adjusting for age at diagnosis and after adjustment for age at diagnosis, SEIFA index, residence, and method of diagnosis, except when adjusting for age at diagnosis alone in patients with lower PSA concentrations at diagnosis.

Table 3 indicates the primary treatments, excluding EBRT, undertaken by PrCa patients treated in the public and private healthcare systems (full list of treatments available in Supplementary Table S2). Amongst men not treated with EBRT, the most common treatments were RP and AS, accounting for over 70% of patients. RP was predominant among publicly treated patients (43.0%), whereas AS was the most frequent option for private patients (52.9%).

Table 4 presents primary treatments stratified by disease risk to restrict comparison to patients with a similar disease state at diagnosis (n = 2038 patients with institute and risk category data). The majority of low-risk patients in both the public and private system were managed with either WW or AS, compared with all other risk categories, where a greater proportion of patients received an active therapy across both healthcare sectors. However, men diagnosed in the public system with intermediate- and very high-risk/metastatic disease were significantly more likely to undergo an active treatment compared to private patients. Chemical ADT, alone or in combination with chemotherapy, was the most common treatment for those diagnosed with very high-risk/metastatic disease.

When opting to undergo active treatment, overall, patients treated in the public health system took significantly longer to commence treatment (Table 5). These differences were present across the low-, intermediate-, and high-risk groups, and persisted after adjustment for age at diagnosis, and after adjustment for age at diagnosis, SEIFA index, residence, and method of diagnosis. Only very high-risk/metastatic patients had a comparable time to commence treatment.

## 4. Discussion

Using clinicopathological and treatment data from PCOR-TAS, our study demonstrated that PrCa patients treated in the Tasmanian public system were older, more likely to reside in outer regional/remote areas of the state, be from a more socioeconomically disadvantaged area, and less likely to be from the most advantaged areas. They were significantly more likely to be diagnosed via TRUS, and less likely to be diagnosed via TP biopsy. Public patients were also more likely to be diagnosed with more advanced disease: men diagnosed in the public system were significantly less likely to be classified as low-risk and were more likely to be diagnosed with high-risk, very high-risk, and metastatic disease. Similarly, te Marvelde et al. (2020) [7] looked at 29,325 Victorian men diagnosed with PrCa and found that men diagnosed in the public system were more socioeconomically disadvantaged, older, had higher grade tumours, and were more likely to reside in areas outside of major cities.

Tasmania represents a wholly regional state of Australia, with over a third of the population residing in rural areas and no major cities present within the state. We know that men living in outer regional or remote areas are significantly more likely to be diagnosed in a public health institution [9]. With all providers of PrCa diagnostic and treatment care located within inner regional postcodes, Tasmanian PrCa patients residing in outer regional and remote locations may face a serious accessibility barrier to care. This is reflected in the time taken to access treatment, and supported by our previous study [9], which showed that men living in rural areas take longer to commence active PrCa treatment, and travel significantly further to do so.

Close to a third of Tasmanian men treated in the public system were diagnosed via TRUS, while a significantly greater proportion of privately treated patients underwent TP biopsy. TP biopsy has been widely considered as best practice, mainly due to observational studies that have shown a lower risk of infection [12] and better cancer detection rates [13,14]. However, recent clinical trials comparing TP to TRUS biopsy have demonstrated no differences in infection rates [15,16] nor in detection rates of clinically significant cancer [17,18], particularly within an MRI-targeted pathway. Thus, the disparity in biopsy methods may not translate into worse outcomes for either patient group. Notably, Tasmanian public healthcare facilities did not have the equipment required for TP biopsy until fairly recently, so the inequities observed in this study cohort may not be relevant to future cohorts.

Outside of EBRT, men treated in the private system were most likely to be managed with AS (52.9%), followed by RP (31.3%) or ADT (8.9%). In contrast, only 29.6% of publicly treated patients were managed with AS. While this disparity could be due to a greater proportion of low-risk patients being diagnosed in the private system, when patients are stratified by disease risk, a similar proportion of low-risk patients are managed with AS across the two health systems. However, there is a significant difference in the proportion of men managed with AS in the intermediate- and very high-risk/metastatic disease groups, where private patients are managed more conservatively. Conversely, Tasmanian public patients are more likely to be managed with active treatment, where the most common treatment, outside of EBRT, was RP (43.0%). As noted above, these differences remained significant in men diagnosed with intermediate- and very high-risk/metastatic disease. Only one previous study, based on ~2700 Victorian participants diagnosed with PrCa, has shown an association between patients being diagnosed in a private hospital and not receiving active treatment [19]. In contrast, results from three other mainland Australian cohorts, indicated that a greater proportion of patients in the private healthcare system underwent RP compared to public patients [7,20,21]. While these studies did not evaluate treatment by place of residence and health service type, te Marvelde et al. (2020) [7] found that even within the lower socio-economic quintiles, which we have shown to be associated with area of residence in the Tasmanian population [9], private patients were still more likely to undergo RP compared to public healthcare patients. While we are yet unable to determine whether these differences in treatment strategies between the public and private healthcare systems impact patient outcomes (due to the PCOR-TAS cohort being relatively “young”), it will be critical to evaluate this in future years.

Interestingly, the overall proportion of men in our study receiving no active treatment (42.8%) was greater than that observed by Evans et al. (2013; 22.7%) [19], a US Medicare-linked study (2007; ~23%) [22], and a South Australian study monitoring men treated in the public system (2009; ~20%) [23]. A study by Wang et al. (2018) [20], based on PCOR-VIC data from 14,025 men, found comparable proportions of patients diagnosed in the public and private sectors received no active treatment, with an overall rate of 24%. The increased rates of AS observed in regional Tasmania may be influenced by greater distances to access treatment and/or limited treatment facilities, thus delaying treatment commencement and/or influencing treatment decisions. Furthermore, our observations are based on treatment within twelve months of diagnosis; many men may have simply taken time to consider their treatment options and chose to undertake active treatment after this 12-month time period.

The differences in Tasmanian public and private hospital treatment strategies have the potential to significantly impact patients. Studies comparing long-term outcomes and quality of life among men with PrCa over up to fifteen years have shown persistent and significant differences based on treatment type. Men who initially manage their PrCa with AS generally do not experience the acute effects of active treatment, such as impaired sexual and urinary function; however, physical wellbeing may decline over time as a result of disease progression, age-related effects, and the commencement of active treatments [24,25]. Multiple studies have found that RP has a substantial negative impact on sexual function, both immediately post-treatment and for multiple years thereafter [24,25,26,27,28]. RP is also known to impact urinary function and cause incontinence [25,28,29], and, notably, it has been reported that nerve-sparing surgical approaches are not protective against urinary incontinence [25]. For men treated with ADT, associations between this therapy and poorer quality of life have been observed [25,26], with Mazariego et al. (2020) [26] finding that patients treated with ADT were the only group to exceed the threshold for significantly impaired mental wellbeing at fifteen years post-diagnosis.

Importantly, not all treatment side effects are regarded equally by those affected. While adverse impacts on sexual function may be most frequently associated with RP, patients have reported that this causes less distress than the less common, but more concerning effect of urinary incontinence [25]. This concurs with a 2012 study, where it was found that the least tolerable side effect of PrCa treatment was urinary leakage, and subsequently concluded that significant survival benefits were needed to offset such adverse treatment effects [30]. Importantly, with time, collection of PCOR-TAS outcomes data will permit an in-depth exploration of how treatment choice affects the quality of life of Tasmanian PrCa patients.

Finally, patients treated in the public system took significantly longer to commence active treatment, compared to those being treated privately, except for patients with very high-risk/metastatic disease. This was true across all remaining disease risk categories, and significance persisted after adjustment for age at diagnosis, SEIFA index, residence, and method of diagnosis. As the registry ages, data will permit the examination of the impact of a delay in commencing treatment on survival and quality of life outcomes in these men.

A notable strength of this study is its leveraging of the existing, Movember-funded PCOR-TAS, which comprises a comprehensive collection of demographic and clinical variables. As such, this study has been able to interrogate data from the regional state of Tasmania to ascertain disparities in patient treatment strategies between the public and private health sectors. Treatment strategies were significantly different between the two sectors, with a higher proportion of private patients being managed with AS, while public patients were more likely to have invasive treatment. The majority of public patients also waited significantly longer to be treated. Study results should be considered in light of several limitations, the most significant of which is that PCOR-TAS data alone did not allow us to determine whether radiotherapy was undertaken in a public or private facility, as a proportion of patients who receive their treatment privately, do so in public facilities. In future, linkage to national Medicare Benefits Scheme data may overcome this issue. We were also not able to stratify RP procedures into open or robot-assisted categories due to limited numbers, as this procedure was only made available in Tasmania in July 2019. Another limitation to consider is the registry age, having only commenced in 2015. As such, there has not been sufficient time to accrue numbers for recurrence and survival outcome analyses. Furthermore, consistent collection of patient-reported outcome measures was only available from 2018 onwards, thus precluding their evaluation in this study.

## 5. Conclusions

There are a range of treatment options available for men with PrCa, with varying impacts on physical and mental health in the long and short term. It is important that information on the risks and benefits of all treatment or disease management strategies appropriate for their disease state is available to patients, and that limited access to healthcare does not influence this decision, as the impacts on quality of life may last decades. Given the higher incidence and mortality rates in regional Australia, insights from this study can guide the development of research-supported procedures for policymakers and healthcare services, ensuring equity of treatment and improving outcomes for all men affected with PrCa.

## Figures and Tables

**Table 1:** Demographic and clinical characteristics of Tasmanian prostate cancer patients by healthcare facility type.

	Public, % (n)		Private, % (n)		*p*
**Included**	31.7%	(690)	68.3%	(1490)	
**Age at Diagnosis**					0.0968
<60	18.7%	(129)	17.3%	(257)	
60–64	17.8%	(123)	18.7%	(278)	
65–69	25.69%	(179)	24.2%	(360)	
70/74	21.0%	(145)	18.7%	(279)	
≥75	16.5%	(114)	21.2%	(316)	
**Age at Diagnosis (Median (IQR))**	67.7	(62.0–72.5)	68.1	(62.5–73.8)	**0.036**
**SEIFA Quintile**					**<0.001**
1 (Most Disadvantaged)	19.3%	(133)	10.9%	(163)	
2	32.8%	(226)	22.0%	(327)	
3	16.4%	(113)	13.8%	(205)	
4	20.1%	(139)	24.7%	(368)	
5 (Most Advantaged)	10.6%	(73)	28.2%	(420)	
Missing	0.9%	(6)	0.5%	(7)	
**Residence**					**<0.001**
Inner Regional	53.0%	(366)	66.8%	(996)	
Outer Regional/Remote	46.1%	(318)	32.7%	(487)	
Missing	0.9%	(6)	0.5%	(7)	
**Method of Diagnosis**					**<0.001**
TRUS	32.5%	(224)	11.5%	(171)	
TURP	10.1%	(70)	14.0%	(208)	
TP Biopsy	55.4%	(382)	73.6%	(1096)	
Other	2.0%	(14)	1.0%	(15)	
**Diagnostic PSA (ng/mL)**					**<0.001**
<4	6.4%	(44)	13.0%	(193)	
4.01–10	46.2%	(319)	56.7%	(845)	
10.01–20	21.5%	(148)	16.6%	(247)	
>20.01	16.2%	(112)	7.3%	(109)	
Missing	9.7%	(67)	6.4%	(96)	
**Gleason Score**					**<0.001**
<7	31.0%	(214)	55.12%	(823)	
7 (3 + 4)	27.0%	(186)	23.4%	(349)	
7 (4 + 3)	16.1%	(111)	9.3%	(138)	
8	12.5%	(86)	5.4%	(80)	
>8	12.8%	(88)	6.4%	(95)	
Missing	0.7%	(5)	0.3%	(5)	
**Disease Risk Category**					**<0.001**
Low	18.1%	(125)	39.1%	(582)	
Intermediate	40.3%	(278)	37.5%	(559)	
High	15.5%	(107)	9.5%	(141)	
Very High	2.0%	(14)	0.9%	(14)	
Metastatic	16.7%	(115)	6.9%	(103)	
Missing	7.4%	(51)	6.1%	(91)	
**Primary Treatment**					**<0.001**
Active Surveillance	29.6%	(204)	52.9%	(788)	
Active Treatment	68.3%	(471)	45.5%	(678)	
Watchful Waiting	1.6%	(11)	0.7%	(10)	
Unspecified WW/AS	0.6%	(4)	0.9%	(13)	
Missing	0%	(0)	0.1%	(1)	
**Days to Active Treatment (Median (IQR))**	119	(79–167)	73	(52–114)	**<0.001**

IQR = Interquartile Range; SEIFA = Socio-Economic Index of Advantage and Disadvantage; TRUS = Transrectal Ultrasound; TURP = Transurethral Resection of the Prostate; TP Biopsy = Transperineal Biopsy; PSA = Prostate Specific Antigen. Significant results are bolded.

**Table 2:** Factors associated with treatment of prostate cancer in a public compared with private Tasmanian healthcare facility.

	Unadjusted, PR (95% CI)		Adjusted *, PR (95% CI)		Adjusted †, PR (95% CI)		Adjusted ‡, PR (95% CI)	
**Age at Diagnosis**								
<60	1.08	(0.90–1.31)						
60–64	0.96	(0.79–1.16)						
65–69	n.a.							
70–74	1.12	(0.94–1.34)						
≥75	**0.78**	**(0.64–0.95)**						
**SEIFA Quintile**								
1 (Most Disadvantaged)	**1.77**	**(1.43–2.18)**	**1.77**	**(1.43–2.18)**				
2	**1.50**	**(1.30–1.73)**	**1.50**	**(1.30–1.73)**				
3	n.a.		n.a.					
4	0.82	(0.69–1.97)	**0.82**	**(0.69–0.97)**				
5 (Most Advantaged)	**0.38**	**(0.30–0.48)**	**0.38**	**(0.30–0.48)**				
**Residence**								
Inner Regional	n.a.		n.a.		n.a.			
Outer Regional/Remote	**1.41**	**(1.28–1.56)**	**1.41**	**(1.28–1.56)**	**1.25**	**(1.19–1.31)**		
**Method of Diagnosis**								
TRUS	**2.83**	**(2.37–3.38)**	**2.78**	**(2.33–3.32)**	**2.54**	**(2.06–3.12)**		
TURP	n.a.		n.a.		n.a.			
TP Biopsy	**0.75**	**(0.70–0.81)**	**0.74**	**(0.69–0.80)**	**0.79**	**(0.73–0.86)**		
Other	2.02	(0.98–4.15)	**2.28**	**(1.13–4.59)**	2.04	(0.87–4.79)		
**Diagnostic PSA (ng/mL)**								
<4	n.a.		n.a.		n.a.			
4.01–10	**0.84**	**(0.77–0.92)**	0.92	(0.84–1.01)	**0.88**	**(0.79–0.98)**		
10.01–20	**1.34**	**(1.12–1.61)**	1.15	(0.94–1.40)	0.99	(0.75–1.26)		
>20.01	**2.30**	**(1.80–2.94)**	**1.76**	**(1.35–2.31)**	**2.45**	**(1.74–3.44)**		
**Gleason Score**								
<7	**0.56**	**(0.50–0.64)**	**0.56**	**(0.50–0.63)**	**0.57**	**(0.50–0.65)**		
7 (3 + 4)	n.a.		n.a.		n.a.			
7 (4 + 3)	**1.74**	**(1.38–2.20)**	**1.74**	**(1.38–2.20)**	**1.70**	**(1.31–2.20)**		
8	**2.33**	**(1.74–3.12)**	**2.34**	**(1.76–3.11)**	**2.13**	**(1.54–2.95)**		
>8	**2.01**	**(1.52–2.65)**	**2.03**	**(1.57–2.63)**	**2.45**	**(1.82–3.31)**		
**Disease Risk Category**								
Low	**0.47**	**(0.40–0.56)**	**0.47**	**(0.40–0.56)**	**0.47**	**(0.39–0.55)**		
Intermediate	n.a.		n.a.		n.a.			
High	**1.66**	**(1.32–2.10)**	**1.68**	**(1.34–2.10)**	**1.56**	**(1.22–2.00)**		
Very High/Metastatic	**2.41**	**(1.91–3.04)**	**2.46**	**(1.97–3.06)**	**2.91**	**(2.26–3.74)**		
**Primary Treatment**								
WW/AS	n.a.		n.a.		n.a.		n.a.	
Active	**1.50**	**(1.39–1.62)**	**1.49**	**(1.39–1.61)**	**1.25**	**(1.19–1.30)**	**1.07**	**(1.03–1.11)**

PR = Prevalence Ratio; CI = Confidence Interval; n.a. = Not Applicable; SEIFA = Socio-Economic Index of Advantage and Disadvantage; TRUS = Transrectal Ultrasound; TURP = Transurethral Resection of the Prostate; TP Biopsy = Transperineal Biopsy; PSA = Prostate Specific Antigen. * Adjusted for age at diagnosis. † Adjusted for age at diagnosis, SEIFA index, residence, and method of diagnosis. ‡ Adjusted for age at diagnosis, SEIFA index, residence, method of diagnosis, and disease risk category. Significant results are bolded.

**Table 3:** Types of primary treatments undertaken by Tasmanian men with prostate cancer.

Treatment	Public, % (n)		Private, % (n)		Missing Institution Data, (n)		Total, % (n)	
Active Surveillance	29.6%	(204)	52.9%	(788)	6.8%	(20)	40.9%	(1012)
ADT (Chemical)	15.2%	(105)	8.9%	(132)	0.3%	(1)	9.6%	(238)
ADT (Surgical)	0%	(0)	0.3%	(4)	0%	(0)	0.2%	(4)
Brachytherapy	1.5%	(10)	2.1%	(31)	1.4%	(4)	1.8%	(45)
Brachytherapy + ADT (Chemical)	0.4%	(3)	0.1%	(2)	0%	(0)	0.2%	(5)
Chemotherapy	0.3%	(2)	0.2%	(3)	0%	(0)	0.2%	(5)
Chemotherapy + ADT (Chemical)	6.2%	(43)	1.3%	(20)	0%	(0)	2.5%	(63)
Focal Gland Ablation Therapy	0%	(0)	0%	(0)	1.0%	(3)	0.1%	(3)
Surgery	43.0%	(297)	31.3%	(467)	48.0%	(142)	36.6%	(906)
Surgery + ADT (Chemical)	1.3%	(9)	1.3%	(19)	1.7%	(5)	1.3%	(33)
Surgery + Chemotherapy + ADT (Chemical)	0.3%	(2)	0%	(0)	0%	(0)	0.1%	(2)
Watchful Waiting	1.6%	(11)	0.7%	(10)	2.4%	(7)	1.1%	(28)
WW/AS Unspecified	0.6%	(4)	0.9%	(13)	0.7%	(2)	0.8%	(19)
Missing	0.1%	(1)	0%	(0)	37.8%	(112)	4.6%	(113)
Total		690		1490		296		2476

ADT = Androgen Deprivation Therapy; WW/AS = Watchful Waiting/Active Surveillance.

**Table 4:** Treatment approaches, stratified by disease risk category, of men with prostate cancer treated in Tasmanian public and private healthcare facilities.

	Low-Risk				Intermediate-Risk				High-Risk				Very High-Risk/Metastatic			
	Public, % (n)		Private, % (n)		Public, % (n)		Private, % (n)		Public, % (n)		Private, % (n)		Public, % (n)		Private, % (n)	
**Surveillance**	82.4%	(103)	84.4%	(491)	24.1%	(67)	39.2%	(219)	15.0%	(16)	17.7%	(25)	0.8%	(1)	8.6%	(10)
**Active**	17.6%	(22)	15.6%	(91)	75.9%	(211)	60.6%	(339)	85.0%	(91)	82.3%	(116)	99.2%	(128)	91.4%	(107)
**Treatment approach:**																
Active Surveillance	81.6%	(102)	82.7%	(481)	23.0%	(64)	37.8%	(211)	10.3%	(11)	17.0%	(24)	0.8%	(1)	8.6%	(10)
Watchful Waiting	0.8%	(1)	0.7%	(4)	1.1%	(3)	0.5%	(3)	2.8%	(3)	0.7%	(1)	n.a.	(0)	n.a.	(0)
WW/AS Unspecified	n.a.	(0)	1.0%	(6)	n.a.	(0)	0.9%	(5)	1.9%	(2)	n.a.	(0)	n.a.	(0)	n.a.	(0)
ADT (Chemical)	n.a.	(0)	0.3%	(2)	0.7%	(2)	3.0%	(17)	25.2%	(27)	29.1%	(41)	56.6%	(73)	59.0%	(69)
ADT (Surgical)	n.a.	(0)	n.a.	(0)	n.a.	(0)	0.2%	(1)	n.a.	(0)	0.7%	(1)	n.a.	(0)	1.7%	(2)
Brachytherapy	3.2%	(4)	1.4%	(8)	2.2%	(6)	3.8%	(21)	n.a.	(0)	n.a.	(0)	n.a.	(0)	n.a.	(0)
Brachytherapy + ADT (Chemical)	0.8%	(1)	n.a.	(0)	0.7%	(2)	0.4%	(2)	n.a.	(0)	n.a.	(0)	n.a.	(0)	n.a.	(0)
Chemotherapy	0.8%	(1)	n.a.	(0)	n.a.	(0)	0.2%	(1)	n.a.	(0)	0.7%	(1)	%	(0)	n.a.	(0)
Chemotherapy + ADT (Chemical)	n.a.	(0)	n.a.	(0)	n.a.	(0)	n.a.	(0)	n.a.	(0)	n.a.	(0)	%	(0)	17.1%	(20)
Focal Gland Ablation Therapy	n.a.	(0)	n.a.	(0)	n.a.	(0)	n.a.	(0)	n.a.	(0)	n.a.	(0)	n.a.	(0)	n.a.	(0)
Surgery	12.0%	(15)	13.8%	(80)	70.9%	(197)	52.8%	(295)	57.9%	(62)	46.1%	(65)	6.2%	(8)	7.7%	(9)
Surgery + ADT (Chemical)	0.8%	(1)	0.2%	(1)	1.4%	(4)	0.4%	(2)	1.9%	(2)	5.7%	(8)	0.6%	(2)	6.0%	(7)
Surgery + Chemotherapy + ADT (Chemical)	n.a.	(0)	n.a.	(0)	n.a.	(0)	n.a.	(0)	n.a.	(0)	n.a.	(0)	0.8%	(1)	n.a.	(0)
Missing	%	(0)	n.a.	(0)	%	(0)	0.2%	(1)	%	(0)	n.a.	(0)	%	(0)	n.a.	(0)
**Total**		125		582		278		559		107		141		129		117
***p* (Surveillance vs. Active)**	0.5866				**<0.001**				0.5598				**0.0032**			

n.a. = Not Applicable; WW/AS = Watchful Waiting/Active Surveillance; ADT = Androgen Deprivation Therapy; Significant results are bolded.

**Table 5:** Differences in time to primary treatment between prostate cancer patients treated in Tasmanian public and private healthcare facilities.

	Time in Days, Median (IQR)		Additional Time for Individuals Treated in Public Healthcare Facilities (n = 2038)					
			Unadjusted, MD (95% CI)		Adjusted *, MD (95% CI)		Adjusted †, MD (95% CI)	
**Time to Primary Active Treatment, Low-Risk (days)**								
Private	107	(61.5–188.5)	n.a.		n.a.		n.a.	
Public	173.5	(90–238)	**52.30**	**(13.62–90.98)**	**51.31**	**(14.03–88.58)**	**43.46**	**(12.19–74.73)**
(n)		(707)						
**Time to Primary Active Treatment, Intermediate-Risk (days)**								
Private	83	(61–119)	n.a.		n.a.		n.a.	
Public	135	(105–185)	**50.79**	**(40.33–61.26)**	**51.91**	**(41.22–62.59)**	**59.22**	**(47.82–70.62)**
(n)		(837)						
**Time to Primary Active Treatment, High-Risk (days)**								
Private	62	(40–86)	n.a.		n.a.		n.a.	
Public	119	(85–158)	**47.79**	**(29.83–64.35)**	**42.22**	**(25.03–59.42)**	**42.25**	**(23.23–61.26)**
(n)		(248)						
**Time to Primary Active Treatment, Very High-Risk/Metastatic (days)**								
Private	42.5	(18.5–72)	n.a.		n.a.		n.a.	
Public	46.5	(22–104)	9.23	(−5.16–23.62)	8.66	(−6.43–23.74)	10.22	(−2.43–22.87)
(n)		(246)						

IQR = Interquartile Range; MD = Mean Difference; CI = Confidence Interval; n.a. = Not Applicable; * Adjusted for age at diagnosis. † Adjusted for age at diagnosis, SEIFA index, residence, and method of diagnosis. Significant results are bolded.

## Data Availability

Access to PCOR-TAS data is guided by strict protocols and procedures in order to ensure that the privacy of all participants is protected at all times and other ethical concerns are considered. Queries regarding the PCOR-TAS data access policy and a data request form can be directed to: pcor.tasmania@utas.edu.au.

## Supplementary Materials

The following supporting information can be downloaded at: https://www.mdpi.com/article/10.3390/cancers18010079/s1, Table S1: PCOR-TAS defined risk categories based on National Comprehensive Cancer Network guidelines and data availability; Table S2: Full list of primary prostate cancer treatment options undertaken by Tasmanian prostate cancer patients; Figure S1: SEIFA quintiles based on Postal Areas (POA) 2021.
