# Supplementary material for: Prostate Cancer Disparities Between Public and Private Healthcare Patients in Tasmania, a Regional State of Australia
Source: Cancers (Basel). 2025 Dec 26;18(1):79. doi: 10.3390/cancers18010079 (PMC12784806; doi:10.3390/cancers18010079)
Supplement: Supplementary file 1 [file cancers-18-00079-s001.zip › cancers-4027177-supplementary.pdf]

Supplementary Table S1 – PCOR-TAS defined risk categories based on National Comprehensive Cancer Network guidelines and data availability*.

Risk Group	NCCN Category	Clinical / Pathological Features
Low	1	T2A or less AND<br>PSA less than 10 ng/mL AND<br>Gleason score less than 7
Intermediate	2	T2b OR T2c OR<br>Gleason score 7 OR<br>PSA 10-20 ng/mL
High	3	T3 OR T3a OR<br>PSA greater than 20 ng/mL OR<br>Gleason score 8 - 10
Very High	4	T3b OR T4 OR T4a OR T4b OR<br>Primary Gleason pattern 5
Metastatic	5	N1 OR M1
Not Stated	-1	No data available

*Note that biopsy information used in the standard NCCN risk classification was not available.

Supplementary Table S2 - Full list of primary prostate cancer treatment options undertaken by Tasmanian prostate cancer patients.

Treatment	Public, % (n)	Private, % (n)	Missing Institution Data, (n)	Total, % (n)
Active Surveillance	29.6% (204)	52.9% (788)	1.7% (20)	30.1% (1,012)
ADT (Chemical)	15.2% (105)	8.9% (132)	0.1% (1)	7.1% (238)
ADT (Surgical)	0% (0)	0.3% (4)	0% (0)	0.1% (4)
Brachytherapy	1.5% (10)	2.1% (31)	0.3% (4)	1.3% (45)
Brachytherapy + ADT (Chemical)	0.4% (3)	0.1% (2)	0% (0)	0.1% (5)
Chemotherapy	0.3% (2)	0.2% (3)	0% (0)	0.1% (5)
Chemotherapy + ADT (Chemical)	6.2% (43)	1.3% (20)	0% (0)	1.9% (63)
Focal Gland Ablation Therapy	0% (0)	0% (0)	0.3% (3)	<0.1% (3)
Surgery	43.0% (297)	31.3% (467)	12.0% (142)	26.9% (906)
Surgery + ADT (Chemical)	1.3% (9)	1.3% (19)	0.4% (5)	1.0% (33)
Surgery + Chemotherapy + ADT (Chemical)	0.3% (2)	0% (0)	0% (0)	<0.1% (2)
Watchful Waiting	1.6% (11)	0.7% (10)	0.6% (7)	0.8% (28)
WW/AS Unspecified	0.6% (4)	0.9% (13)	0.2% (2)	0.6% (19)
Radiotherapy	0% (0)	0% (0)	27.1% (320)	9.5% (320)
Radiotherapy + ADT (Chemical)	0% (0)	0% (0)	37.7% (446)	13.3% (446)
Radiotherapy + ADT (Surgical)	0% (0)	0% (0)	0.1% (1)	<0.1% (1)
Radiotherapy + ADT (Chemical) + WW/AS	0% (0)	0% (0)	0.4% (5)	0.1% (5)
Radiotherapy + ADT (Chemical) + Other Systemic Therapies	0% (0)	0% (0)	0.1% (1)	<0.1% (1)
Radiotherapy + Brachytherapy	0% (0)	0% (0)	0.1% (1)	<0.1% (1)
Radiotherapy + Brachytherapy + ADT (Chemical)	0% (0)	0% (0)	0.3% (4)	0.1% (4)
Radiotherapy + Chemotherapy	0% (0)	0% (0)	0.1% (1)	<0.1% (1)
Radiotherapy + Chemotherapy + ADT (Chemical)	0% (0)	0% (0)	1.2% (14)	0.4% (14)
Radiotherapy + Surgery	0% (0)	0% (0)	2.1% (25)	0.7% (25)
Radiotherapy + Surgery + ADT (Chemical)	0% (0)	0% (0)	3.1% (37)	1.1% (37)

Radiotherapy + Surgery + WWAS	0% (0)	0% (0)	0.1% (1)	<0.1% (1)
Radiotherapy + WW/AS	0% (0)	0% (0)	0.9% (11)	1.6% (11)
Radiotherapy + Referred	0% (0)	0% (0)	0.1% (1)	<0.1% (1)
Radiotherapy + Unknown	0% (0)	0% (0)	0.1% (1)	<0.1% (1)
Unknown Combination, including Radiotherapy	0% (0)	0% (0)	1.4% (17)	0.5 (17)
Missing	0.1% (1)	0% (0)	9.5% (112)	3.3% (113)
**Total**	**690**	**1,490**	**1,182**	**3,362**

n.a. = Not Applicable; ADT = Androgen Deprivation Therapy; WW/AS = Watchful Waiting/Active Surveillance.

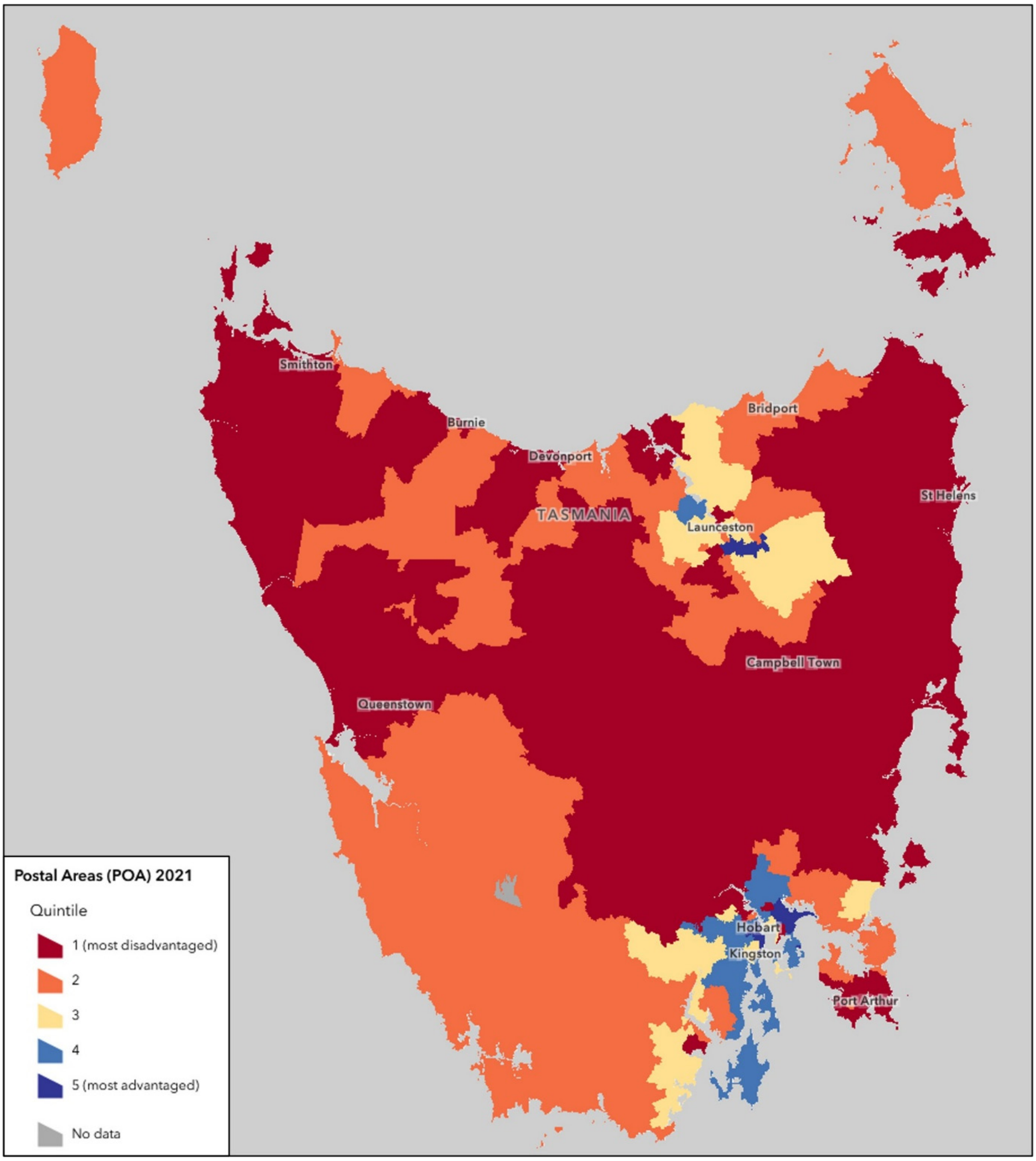

Supplementary Figure S1. SEIFA quintiles based on Postal Areas (POA) 2021. Taken from the Census of Population and Housing: Socio-Economic Indexes for Areas (SEIFA), Australia, 2021. https://experience.arcgis.com/experience/32dcbb18c1d24f4aa89caf680413c741/
